# Probing the Free Volume in Polymers by Means of Positron Annihilation Lifetime Spectroscopy

**DOI:** 10.3390/polym15143128

**Published:** 2023-07-23

**Authors:** Giovanni Consolati, Dario Nichetti, Fiorenza Quasso

**Affiliations:** 1 Department of Aerospace Science and Technology, Politecnico di Milano, Via LaMasa, 34, 20156 Milano, Italy; fiorenza.quasso@polimi.it; 2 INFN, Sezione di Milano, Via Celoria, 16, 20133 Milano, Italy; 3 Rheonic Lab, Via Quadelle 2C, 26012 Castelleone, Italy; dario.nichetti@rheonicsrl.com

**Keywords:** free volume, positron annihilation lifetime spectroscopy, polymers, membranes, biopolymers, composites, rubbers

## Abstract

Positron annihilation lifetime spectroscopy (PALS) is a valuable technique to investigate defects in solids, such as vacancy clusters and grain boundaries in metals and alloys, as well as lattice imperfections in semiconductors. Positron spectroscopy is able to reveal the size, structure and concentration of vacancies with a sensitivity of 10^−7^. In the field of porous and amorphous systems, PALS can probe cavities in the range from a few tenths up to several tens of nm. In the case of polymers, PALS is one of the few techniques able to give information on the holes forming the free volume. This quantity, which cannot be measured with macroscopic techniques, is correlated to important mechanical, thermal, and transport properties of polymers. It can be deduced theoretically by applying suitable equations of state derived by cell models, and PALS supplies a quantitative measure of the free volume by probing the corresponding sub-nanometric holes. The system used is positronium (Ps), an unstable atom formed by a positron and an electron, whose lifetime can be related to the typical size of the holes. When analyzed in terms of continuous lifetimes, the positron annihilation spectrum allows one to gain insight into the distribution of the free volume holes, an almost unique feature of this technique. The present paper is an overview of PALS, addressed in particular to readers not familiar with this technique, with emphasis on the experimental aspects. After a general introduction on free volume, positronium, and the experimental apparatus needed to acquire the corresponding lifetime, some of the recent results obtained by various groups will be shown, highlighting the connections between the free volume as probed by PALS and structural properties of the investigated materials.

## 1. Introduction

Although not univocally defined [1], the concept of free volume can be used to explain various features of polymers. For instance, mechanical properties [2] are generally (negatively) correlated to the free volume fraction of the polymer. Indeed, the applied load tends to concentrate in the free volume instead of distributing among the molecules of the polymer, and this can result in the failure of the material. Therefore, a polymer with a reduced free volume fraction generally shows better mechanical properties [3]. Transport properties are of the utmost importance in polymer membranes used in molecular separation processes such as water desalinization or environmental remediation. In this case, too, the free volume holes in the polymer matrix are responsible for gas separation [4,5]. Another feature involving free volume is physical aging [6]: an amorphous polymer exposed to temperatures below the glass transition for prolonged periods of time shows an increment of the mass density, with decrement of the molecular configurational energy (enthalpy relaxation). When the polymer is cooled from temperatures sufficiently high, the relationship between specific volume and temperature is linear and the structure is in energetic equilibrium. Continuing to cool the polymer, a deviation from linearity is noted in a restricted temperature range: on passing from the rubbery to the glassy state, the specific volume decreases at a rate lower than above the glass transition. The curve of the specific volume shrinkage runs parallel (but higher) to that of the same material ideally crystalline at equilibrium and resulting from the thermal contraction. This behavior is explained in terms of a reduction in the free volume. Struik [7] defined the physical aging using the concept of free volume, showing that large mechanical deformations can result in rejuvenation of the glass. Furthermore, a fundamental linear correlation between the glass transition temperature and free volume fraction has been derived at the molecular level [8]. Roughly speaking, the free volume is the difference between the total volume and ‘occupied’ volume. The different possible definitions of the latter [9] bring about the ambiguity of the idea of free volume. Indeed, when the occupied volume is formulated in terms of the Van der Waals volume [9], the free volume attains the maximum limiting value. On the other hand, in other models [10] the occupied volume also comprises the volume swept out by a molecular segment due to thermal vibration (‘fluctuation volume’ [9]). The difference between the total volume and this ‘vibrational’ volume is excess free volume, which allows movements of polymer segments. Free volume holes refer to this excess free volume, which has been incorporated in some cell models [11].

Holes can be examined by various probes, such as photochromic labels [12], fluorescence molecules [13], or techniques such as small angle XRD [14]. Positron annihilation lifetime spectroscopy (PALS) uses positronium (Ps), a bound electron-positron state, to gain information about the size and distribution of free volume holes (information about the physical properties of Ps can be found in a review by Berko and Pendleton [15]). This non-destructive technique is based on the fact that Ps is repelled from the molecules of the polymer, due to the exchange repulsion between the Ps electron and surrounding electrons and localizes into the open spaces of the host structure. In this sense it is a ‘seeker’ of free volume holes. Since these last have unequal sizes, the probability that Ps tunnels from the hole where it is confined to neighboring holes is negligible [16]. Therefore, Ps can effectively probe the size of the holes, as long as its lifetime is less than that of the cavity hosting it. Ps is a suitable probe even for small cavities, since its size is the same as hydrogen; however, it is much lighter (by a factor of about 2000). As a consequence, quantum effects have to be taken into consideration.

Positron annihilation techniques are not limited to polymer systems, but have been widely used in almost every field of materials science, e.g., to study and identify lattice imperfections in semiconductors [17,18] and metals [19]; understand structural changes occurring in age-hardenable alloys [20]; investigate damages induced by neutron irradiation [21] and electron irradiation, [22] as well as ion implantation [23], to get insight into defects formed in ceramics [24] and explore surfaces [25].

Since this paper is mainly addressed to readers not familiar with the PALS technique, in the following we will introduce the main features of Ps formed in polymers. In the light of the fact that excellent reviews have been published in the past [26,27], we will concentrate only on the recent literature, and on some of the results obtained by our research group. Due to space limitations, we could not quote all the published papers, and the choice reflects our personal interests and aim to point out positron results correlated to structural properties of the examined materials. We apologize for missing references to many interesting works. Although another positron technique, based on the Doppler broadening of the annihilation radiation, is outside the focus of the present study, we remark that it also gives information, although more qualitative, on the free volume [28].

## 2. Positronium in Polymers

Ps is an unstable system, subjected to annihilation. Para-Ps (p-Ps, antiparallel spins) and ortho-Ps (o-Ps, parallel spins) are the two sublevels of ground state Ps, characterized by the different spin orientation of the two particles [29]. In a vacuum, p-Ps has a lifetime of 125 ps; o-Ps lifetime is much longer, 142 ns. In a material, p-Ps is scarcely influenced by the environment and changes in its lifetime are small; on the other hand, in a hole, o-Ps undergoes interactions with surrounding electrons and o-Ps can annihilate, in addition with its own electron, also with an outer electron in relative singlet spin state. This process is called ‘pickoff’, and it is responsible for the decrease in o-Ps lifetime with respect to its value in vacuo up to two orders of magnitude, depending on the overlap between the wave functions of the positron and surrounding electrons [30].

The free volume hole size can be estimated from o-Ps lifetime as supplied by the experiment; to this purpose holes have to be modeled within a suitable geometry. This necessary step allows one to convert the raw results of a measurement into quantitative information. In fact, many experimental techniques use conventional geometries: for instance, in porosimetry, pores are often approximated to cylinders. Concerning PALS, the most popular model for polymers is the spherical one [31,32], although other geometries have been applied, e.g., ellipsoidal cavities [33] have been used to frame the free volume holes in semicrystalline polymers subjected to tensile deformation. Starting from different geometries for the holes, differences in sizes (of the order of 20–30%) are obtained for the same o-Ps lifetime [34].

Tao [31] and later Eldrup [32] found a relationship between o-Ps lifetime and the size of the cavity hosting Ps. Their model assumes a spherical void with effective radius *R*. This Ps trap has a potential well with finite depth; however, for convenience of calculation, an infinite depth is assumed, with a corresponding increase in the radius to *R* + *ΔR*, *ΔR* being an empirical parameter [35] that describes the penetration of the Ps wave function into the bulk. The electron density is zero for *r* < *R* and constant for *r* > *R*. The probability *p* to find Ps inside the bulk polymer is:

(1) $$p=4\pi \int_{R+\Delta R}^{\infty }{\left|\psi \left(r\right)\right|}^{2}r^{2}dr=\frac{\Delta R}{R+\Delta R}+\frac{1}{2\pi }\sin\left(\frac{2\pi R}{R+\Delta R}\right)$$

where [36]:

(2) $$\psi \left(r\right)=\frac{1}{\sqrt{2\pi \left(R+\Delta R\right)}}\frac{\sin\left(\frac{\pi r}{R+\Delta R}\right)}{r}$$

inside the well and zero outside.

The annihilation rate of o-Ps in the bulk state is $\lambda_{0}≅2\;$ ns^−1^, that is, the spin-averaged annihilation rate of p-Ps (8 ns^−1^) and o-Ps (0.007 ns^−1^) in a vacuum. The o-Ps pickoff decay rate $\lambda_{p}$ (ns^−1^) is therefore *λ*_0_ *p* and the relationship with the hole radius *R* is:

(3) $$\lambda_{p}=\lambda_{0}\left[\frac{\Delta R}{R+\Delta R}+\frac{1}{2\pi }sin\left(2\pi \frac{R}{R+\Delta R}\right)\right]$$

The o-Ps lifetime *τ*_3_ as determined by the experiment is the reciprocal of the pickoff decay rate, if we neglect the contribution of the intrinsic decay rate *λ_i_* = 1/142 ns^−1^, which is typically two orders of magnitude less than $\lambda_{p}$. Equation (3) is almost universally adopted to infer the average size of the holes. However, the irregular shape of real holes makes it appropriate to ask whether the spherical model is the most suitable to deduce: (a) the sizes of the holes; (b) the variation in free volume fraction versus a physical variable, such as the temperature.

Another parameter that is available from PALS measurements is o-Ps intensity *I*_3_, which represents the normalized amount of o-Ps in the positron annihilation lifetime spectrum.

It is usually related to the number density of holes, *N*, in the sense that a linear relation between *N* and o-Ps intensity *I*_3_ is assumed [37]. Accordingly, in most of the studies the free volume fraction is written as:

*f* = *CI*_3_*v_h_* (4)

where *v_h_* is the volume of the spherical hole as obtained by o-Ps lifetime using the Tao-Eldrup Equation (3) and *C* is a structural constant. Often a relative free volume, *I*_3_*v_h_*, is used in the discussion of the results. We will come back to this guess later, by discussing some of the results obtained by our group.

In the following Table 1, the lifetimes and intensities of some polymers are shown, together with the corresponding sizes of free volume holes, in spherical approximation. Sometimes, two long components corresponding to Ps in bigger and smaller holes are found.

## 3. Experimental Setup

In most of the studies of bulk polymers, positrons are generated from the decay of ^22^Na, a nuclide emitting a positron with a continuous energy range up to 542 keV and, almost simultaneously, a γ-ray whose energy is 1.274 MeV. This ‘start’ photon signals the birth of the positron. The ‘stop’ signal is provided by one of the annihilation photons: most of the annihilations ordinarily occur into two γ-rays (0.51 MeV each). The source (whose activity is often in the range 0.04 to 1 MBq) is prepared by depositing a droplet of an aqueous solution containing ^22^Na on a thin metallic or plastic foil; after drying, it is covered by an identical support and sealed, to obtain a source that can be used several times. A popular support is the polymide Kapton^®^: it is tough to radiation and Ps does not form in it [49]. The source is inserted between two layers of the sample under study. The thickness of this last should be able to stop 99.9 % of the injected positrons (the range of positrons in matter from a ^22^Na source is about 170 mg/cm^2^) [50].

In order to analyze surfaces and multilayer structures, positrons with lower energies are required. To this purpose, a variable monoenergetic slow positron beam is used, with controlled positron energy from a few eV to a few tens of keV [51,52]. The tuning of the positron energy in such that an apparatus allows one to control the mean implantation depth in the sample. Generally, the positron beam is coupled with a Doppler broadening energy spectrometer; the system supplies valuable information on the layer structure in a multi-layered material [53,54], although PALS can also be used [55,56].

**Figure 1:**
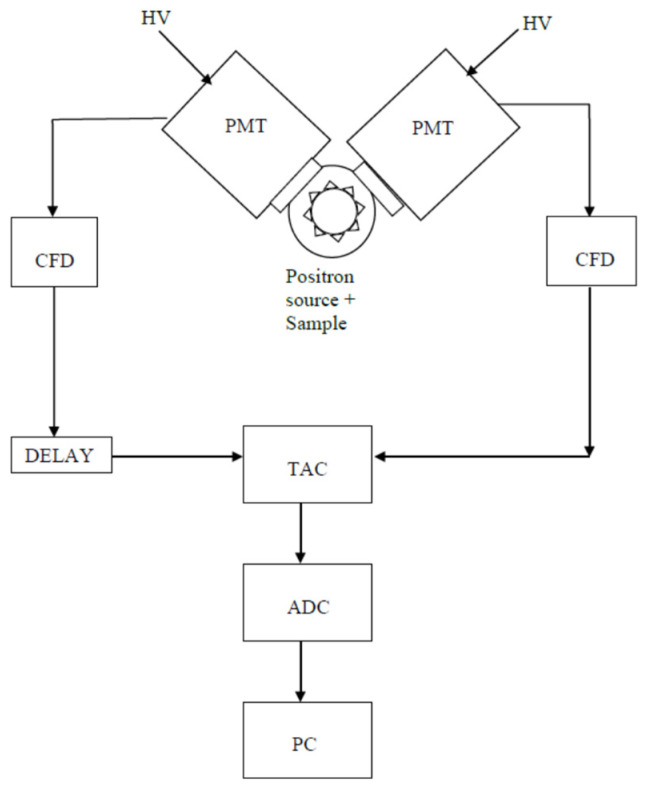
A PALS spectrometer.

Figure 1 is a sketch of a PALS timing spectrometer [57], showing two channels: each one consists of a scintillator coupled to a photomultiplier tube (PMT). The scintillator converts the γ-rays into UV-vis photons, which when hitting the PMT photocathode generates photoelectrons. The most commonly used scintillators are organic (plastic) or fast inorganic (BaF_2_). A constant fraction discriminator (CFD) on each channel generates a fast-timing signal whenever a γ-ray with the correct energy (start or stop) is detected. A time-to-amplitude converter (TAC) activated by the start CFD produces a voltage linearly increasing with time and terminated at the arrival of the stop signal from the other CFD, so that the signal from the TAC is proportional to the lifespan of the positron. This signal, digitized by an analog-to-digital converter (ADC), is transferred to a personal computer (PC). Recently, silicon photomultipliers (SiPM) have been successfully used in the place of traditional PMT [58,59].

**Figure 2:**
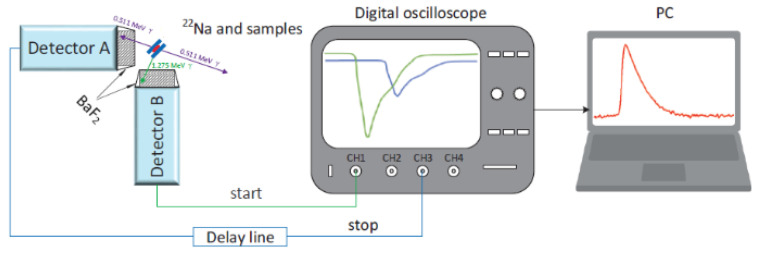
Scheme diagram of the digital positron annihilation lifetime spectrometer. On the oscilloscope, the traces of the 0.511 MeV and 1.27 MeV γ-rays are shown (reproduced from ref. [63] with permission from the Institute of Physics. DOI: 10.1088/1748-0221/15/06/P06001).

Starting from 2000, signals from the PMT can also be digitized by means of ultra-fast modules, replacing some of the units (CFD and TAC) of the apparatus [60,61]. Digitized pulses stored in a personal computer can be analyzed off-line; digital filters select pulses with suitable shape and amplitude (Figure 2). Time resolution is improved with respect to the standard configuration, without decreasing the counting rate [62]. The introduction of ultra-fast digitizers has represented a real milestone in the PALS technique [63,64,65].

The annihilation lifetime spectrum is a histogram (with a statistic of one to several millions of counts), which can be analyzed by means of a suitable computer code. The experimental spectrum results from the convolution of the intrinsic spectrum with a resolution function, which represents the response of the apparatus to two simultaneous events. This can be obtained [66] from the time spectrum of ^60^Co, decaying by emission of two gamma rays with similar energies (1.33 and 1.17 MeV) within a time interval of less than 1 ps, that is, simultaneous in the typical time scale of PALS. Values between 150 and 350 ps can be found for the resolution function.

Several computer programs have been published and made available [67,68,69,70] to analyze the annihilation lifetime spectrum. A PALS spectrum consists of the sum of a number of components, each corresponding to a particular positron state, considered as *discrete* and/or *continuous*. In the first case, a component is an exponential function of the form $\frac{I}{\tau }exp\left(-t/\tau \right)$, characterized by a lifetime *τ* and intensity *I*. The intrinsic spectrum *S*(*t*) can be written:

(5) $$S\left(t\right)=R\left(t\right)⊗\left[\overset{N}{\underset{i=1}{\sum }}\frac{I_{i}}{\tau_{i}}exp(-t/\tau_{i})+B\right]$$

*R*(*t*) being the resolution function and *B* is the background, formed by the spurious coincidence events, which are subtracted during the fitting procedure. The symbol ⊗ represents the convolution operation. Analyses in terms of three components are quite common. A value around 0.4 ns is typical for lifetimes of free positrons (i.e., not forming Ps) in polymers. p-Ps lifetimes in condensed matter are generally within 0.15 ns; o-Ps shows the longest lifetimes, typically in the range 1–10 ns. Sometimes two long components are found in polymer spectra (see Table 1).

A continuous PALS component is built as a continuous sum of discrete components. Three parameters describe it: the intensity and first two moments of the distribution of lifetimes: centroid and second moment (standard deviation from the mean lifetime). A distribution of o-Ps lifetimes (sometimes two distributions) can be expected in a polymeric material, reflecting the hole volume distribution present in the amorphous zones. Both MELT [69] and CONTIN [68] analyze the time annihilation spectrum in terms of continuous components only, without any assumption about the shape of the distributions. The code PALSFIT [67] supplies analyses in term of discrete components. The program LT [70] is also able to give distributions of lifetimes (but assuming a log-normal distribution) and can be used for a ‘mixed’ analysis: each component can be chosen to be either continuous or discrete.

In order to resolve the continuous components of a spectrum with good accuracy, statistics should be higher than a discrete analysis.

Parameters characterizing each component (intensity, lifetime, and standard deviation in the case of distributions) are displayed at the output of each program with the associated statistical uncertainty. Furthermore, the result of a statistical test (e.g., chi square) is given, quantifying the goodness of fit.

## 4. Discussion of Some PALS Results in Polymeric Systems

### 4.1. Membranes

Membranes are the subject of a large number of investigations, due to the obvious interest in many fields. PALS has been applied to different membranes. Considering proton exchange membranes, hybrid Nafion membranes were investigated at different humidities [71]. The authors found that o-Ps lifetime is associated with the microstructure evolution and the development of hydrophilic ion clusters as a function of water uptake. In particular, the maximum value of o-Ps lifetime corresponds to the formation of numerous water channels for proton transportation. This occurs at lower relative humidity for hybrid membranes compared to the pristine one, suggesting that transportation of protons in hybrid membranes can be more efficient at lower relative humidity.

Another investigation concerning two commercial Nafion membranes [72] has pointed out a good correlation between the size of free volume holes as obtained from PALS and proton conductivity, showing that this last is essentially governed by the free volume present in the polymer.

**Figure 3:**
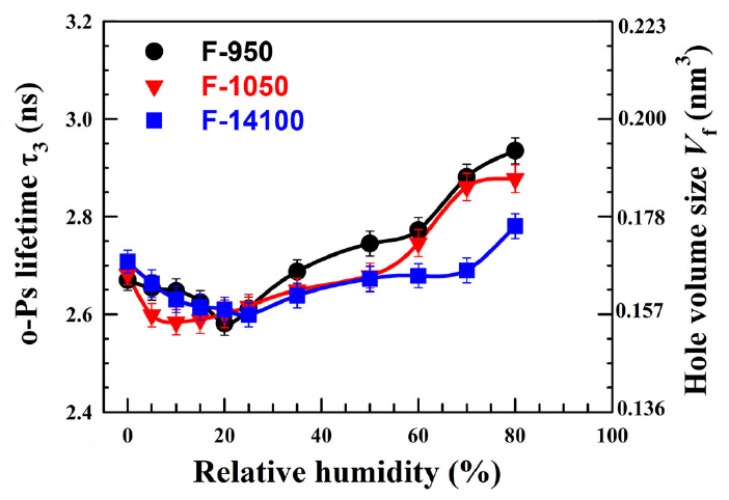
Variation of the o-Ps lifetime as a function of RH for the three investigated membranes. The right *y* axis represents the hole volume size as derived from the Tao-Eldrup model (reproduced from ref. [73] with permission from John Wiley & Sons. DOI: 10.1002/pat5570).

In another study on three commercial per-fluorinated sulfonic acid/PTFE proton exchange membranes [73] similar to Nafion, o-Ps lifetime was studied as a function of time at 70% RH and versus RH, in both cases at room temperature. o-Ps lifetime increases with time, leveling off after about 60 h, which is explained in terms of accumulation of water molecules inside the membranes. Indeed, when the membrane is saturated with water, its nanostructure is stabilized and the hole volume does not change any more. The behavior of o-Ps lifetime with RH is non-monotonous (Figure 3): by increasing RH, lifetime first decreases due to the filling of hole volumes by water molecules. At RH > 20% o-Ps lifetime begins to increase; this is interpreted in terms of membrane plasticization induced by higher water content, which involves an increased hole volume.

As an alternative to Nafion, poly (vinyl alcohol) (PVA) with the added sulfosuccinic acid (SSA) group as a crosslinker is considered as a possible polymeric electrolyte membrane. Indeed, with respect to pure PVA, an improvement in thermal stability, mechanical properties, water uptake, and ion exchange capacity is found. Awad et al. [74] investigated PVA/SSA membranes with different concentrations of SSA. From PALS data, they deduced an increased thermal expansion coefficient of the free volume at increasing content of SSA, both below and above the glass transition. It is interesting to observe that these authors do not correlate the number density of free volume holes to o-Ps intensity, but the decrease in this quantity at increasing SSA concentration is explained in terms of the presence of the SO_3_ group acting as an inhibitor of Ps formation. Indeed, the polar group can capture some of the free electrons created in the terminal track of the thermalizing positron, decreasing the probability of Ps formation.

**Figure 4:**
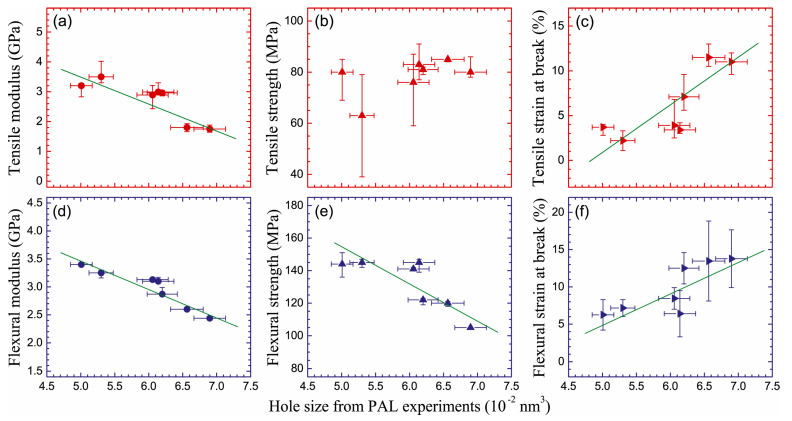
Variations of (**a**) tensile modulus, (**b**) tensile strength, (**c**) tensile strain at break, (**d**) flexural modulus, (**e**) flexural strength, and (**f**) flexural strain at break as functions of hole volume in the seven investigated epoxy resins (reproduced from ref. [76] with permission from Elsevier. DOI: 10.1016/j.polymer.2020.122225).

Another study on crosslinked PVA/SSA proton exchange membranes was undertaken [75], to study the effect of different concentrations of sulfosuccinic acid (SSA) on the properties of PVA membranes. Free volume as determined by PALS supplies a microscopic interpretation of the tensile strength versus the SSA content, which decreases above 15 wt%. Indeed, o-Ps lifetime increases when SSA content > 15 wt%: bigger free volume holes reduce the interactions among the polymer segments, and the corresponding higher chain mobility gives flexibility to the structure, decreasing the tensile strength. A similar conclusion was reached in a study concerning seven chemically different amine-cured epoxy resins [76]: a negative correlation between tensile modulus/flexural modulus and hole volume has been shown, while tensile/flexural strain at break are positively correlated with the hole volume (Figure 4). This allowed the authors to conclude that smaller hole sizes correspond to better tensile and flexural mechanical properties.

Coming back to the study on PVA/SSA membranes [75], the behavior of o-Ps lifetime versus the hydration times in PVA with 30 wt% SSA, initially dried and then saturated at 80% humidity, revealed an expansion of the hole volumes up to about 14 h, leveling off afterwards. Furthermore, a non-monotonic behavior of o-Ps at different relative humidity (RH) was also observed, in the sense that it slightly decreases up to RH 30%, then it increases, in all the membranes with different SSA content. The results are similar to those found in the study concerning the per-fluorinated sulfonic acid/PTFE proton exchange membranes, already quoted [73], with analogous interpretation: the decrease in the free volume holes is explained in terms of occupancy of the intermolecular spaces by water molecules. Higher water uptake involves a strong bonding between the water molecules and hydroxide groups of the structure, inducing softening of the membranes with consequent larger holes.

Polyethylene and Nafion membranes implanted by Cu ions at various fluences (up to 10^17^ ions/cm^2^) were studied using two positron techniques: lifetime and coincidence Doppler broadening (CBD) [77]. Although the polyethylene spectrum is more complex, due to the presence of a Ps component localized in the crystalline zones, in the amorphous phase (predominant in Nafion), the behavior of Ps is similar in the two membranes: at low implantation doses, the free volume holes shrink, which is interpreted by the authors in terms of crosslinking and aggregation of ions in metallic microstructures. By increasing the dose, chain scissions become predominant and free volumes with higher size are created, as probed by an increased o-Ps lifetime.

Poly (vinyl alcohol) (PVA)/graphene oxide (GO) nanocomposite membranes have been the subject of a study [78] aiming to investigate the factors affecting the water vapor transmission rates. The authors found that insertion of nanoplatelets, with corresponding induced tortuous paths, is a major factor but, using PALS, they showed that fractional free volume also plays an important role influencing the water vapor transmission rates.

PALS and the fractional rejection (FR) method were used on cellulose triacetate (CTA) and polyamide (PA) forward osmosis (FO) membranes, in order to investigate the relationship between membrane pores and separation performance [79]. Due to the small thickness of the membranes, a slow positron beam was used. The results show a significantly larger (0.29–0.30 nm) and uniform pore size distribution in both the membranes throughout the active layer depth as compared to typical seawater reverse osmosis membranes (0.20–0.24 nm). Using the FR method, it was found that the mean pore radius of both the membranes was significantly reduced from that obtained using PALS, up to one third in the case of the highest pressures. This is reasonable, taking the compression of thin FO membranes including the active layer into account.

Tetracycline-imprinted membranes prepared using radiation-induced polymerization were investigated by PALS [80] in order to assess the effect of the presence of the template molecule on the size and distribution of free-volume holes. The density of the free volume holes in the imprinted network shows a significant increase due to the presence of the template molecule. Two o-Ps lifetimes were found, attributed to network pores and aggregate pores, with larger size and correspondingly longer lifetime. Transformation of the longest lifetime into hole size gives a value in good agreement with the diameter of tetracycline. In another study on PP/PE non-woven fabrics imprinted by atrazine [81], a similar agreement was found between the typical size of free volume holes and the atrazine diameter.

### 4.2. Composites

**Figure 5:**
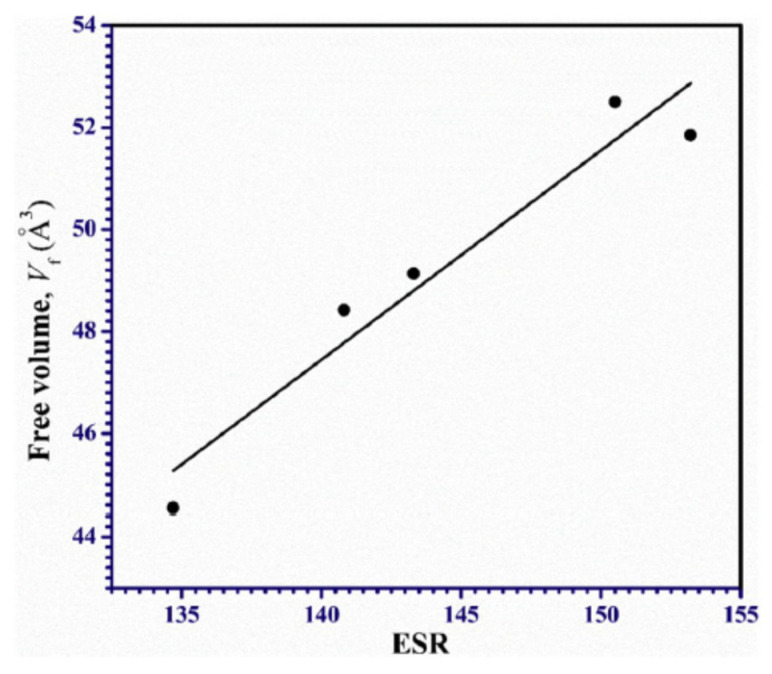
Correlation between hole volume and ESR for the investigated PVA-PVP blends (reproduced from ref. [82] with permission from Elsevier. DOI: 10.1016/j.polymertesting.2020.106681).

Polymer composites are an active research field. PVA-PVP blends were investigated at increasing loading of magnesia, 0.5 up to 2 wt% [82]. o-Ps lifetime is found to decrease with loading magnesia, while intensity remains almost constant. On the whole, the relative fractional free volume, *v_h_I*_3_ decreases, which is interpreted in terms of partially filling the holes in the blend matrix by the magnesia nanoribbons. This is confirmed by a linear relationship between the hole volume and equilibrium swelling ratio, ESR (Figure 5). A correlation can be expected, since any process hindering the water uptake decreases the ESR and magnesia nanoparticles, by decreasing the hole volume available, hamper water absorption.

A study on PDMS filled by fumed silica (FS) was undertaken [83], in order to understand the mechanism of water diffusion after corona discharge, a proxy of the failure of this composite used as insulating material in power grids. PALS was used together with various techniques. A different behavior of o-Ps lifetime was found by increasing the FS content: above about 6% the lifetime, which is stable for lower values, linearly decreases. This is interpreted in terms of a change from a dispersed to a percolated state of the FS in the composite: particles are no longer randomly distributed but tightly packed to the matrix. In this case, the overlapping of FS/PDMS interfaces (which represent extra channels for o-Ps trapping, in addition to the free volume holes) supply continuous diffusion paths for water. This provides a microscopic interpretation of the failure mechanism.

A carbon fiber reinforced polymer consisting of a bisphenol-A epoxy resin and PAN-based carbon fibers were investigated by PALS and X-ray computed tomography, in order to elucidate the microstructural changes occurring during fracture [84]. In situ measurements of X-CT during a tensile test allowed to observe the crack initiation and propagation in the sample. Analysis of o-Ps lifetimes as obtained from the PALS spectra enabled to evaluate free volume hole sizes (0.70 nm) greater than those found in the polymer matrix (0.49 nm) not subjected to testing. These bigger cavities are associated with breaks in the molecular chains upon fracture and result from an agglomeration of holes.

A nanocomposite is a hybrid system with a polymer matrix and filler composed by nanoparticles. A huge number of nanofillers is used, e.g., based on carbon, silica, or metals. Complex and interesting features concern the free volume in the polymeric matrix of the nanocomposite. In fact, while various investigations have shown a decrease in the free volume when the matrix incorporates the nanoparticles (e.g., [85,86,87,88]), in other studies [89,90,91], the free volume increases with the concentration of nanofiller, which has been correlated to a weak interaction between the matrix and the filler, favoring the creation of large free volume holes. On the other hand, an interfacial interaction between polymer matrix and nanofiller sufficiently strong to prevail on the interactions among nanoparticles improves the dispersion of nanofiller in the matrix, with a consequent reduction of free volume. In these cases, an improvement of the tensile properties of the nanocomposite is observed, as it can be expected.

Sometimes, the decrease in the free volume is inverted after a critical concentration of nanofiller. This can be explained in terms of aggregation of nanoparticles: below a critical loading, the interfacial surface between matrix and filler increases and the available free volume decreases. Above a critical loading, aggregation of nanoparticles reduces the interfacial surface and the free volume may increase, even if it can remain below the value of the pristine polymeric matrix [86,92]. Another factor that has to be taken into account is the distribution of free volume holes in the matrix, which is influenced by the presence of nanofillers [93]. In the nanocomposite, this distribution can get narrower [93], for suitable concentration of filler, or can change from a unimodal to a bimodal [94,95].

An exhaustive review of PALS studies in nanocomposites can be found in ref. [27].

### 4.3. Porous Polymers

Micro and mesopores present in polymer networks are studied through gas absorption; comparison with PALS reveals interesting features. For instance, in a series of amorphous melamine-based polymers [96] both PALS and N_2_ absorption isotherms show the same kind of voids; however, PALS gives the correct micropore volume, which is underestimated by the other classical method.

**Figure 6:**
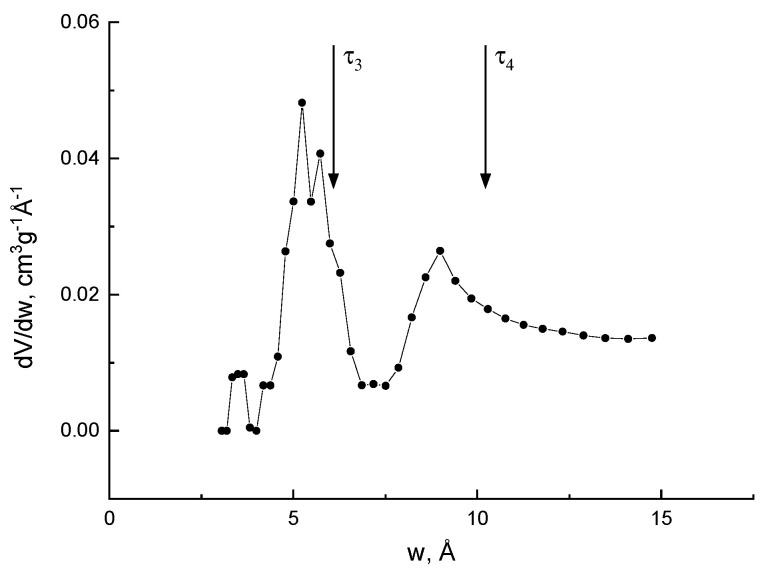
Pore size distribution obtained for a polymer with intrinsic microporosity by the sorption technique with processing, according to the density functional theory NLDFT (*w*: hole size). The vertical arrows indicate the positions of the maxima in the pore size distribution dV/d*w*, according to the positron annihilation data (courtesy by prof. Victor Shantarovich).

Micropores distributions in different amorphous polymer glasses have also been investigated [97] using low-temperature CO_2_ gas sorption and PALS. By processing the data using both the nonlocal density functional theory and Monte Carlo methods, the authors found deviations from a unimodal size distribution. Two o-Ps lifetimes are found and the corresponding effective sizes of the nanopores are very near to the maxima of the distributions found by the sorption technique (Figure 6). Thus, PALS can be a useful tool to study the porosity in amorphous polymers, although limited to microporosity, changes in meso pores (due to e.g., supercritical CO_2_ or aging) being hardly detectable [98].

Polynorbornenes (characterized by various substituent groups in the side chain) have been also investigated using the same techniques [99]. Results from low-temperature sorption of CO_2_ suggest in all the samples bimodal microporosity distributions, while PALS reveals bimodal but also unimodal distributions for low permeability norbornenes. A possible explanation is that the theories applied to describe the sorption curves for low-permeability polymers are not suitable enough. Alternatively, a non-localized positronium could not have sufficient time to find larger pores (that is, more distant from each other) during its lifespan before annihilation. Present investigations do not allow to discriminate between the two possible explanations.

**Figure 7:**
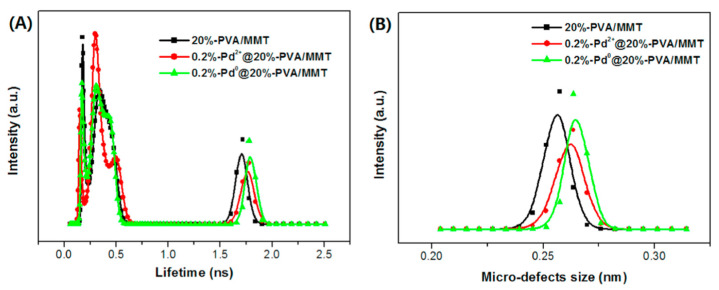
The distribution of positron annihilation lifetime (**A**) and calculated micro-defects size (**B**) of 20%-PVA/MMT matrices and 0.2%-Pd@ 20%-PVA/MMT catalytic composite (reproduced from ref. [100] with permission from Elsevier. DOI: 10.1016/j.radphyschem.2018.09.026).

The microscopic structure of PVA intercalated montmorillonite was studied by Zhao et al. [100]. o-Ps atoms, distributed between the interlayer spacing of MMT as well as in the free volumes of PVA, show a decreasing lifetime as more PVA chains (up to 20%) are intercalated into MMT layers. This is reasonable, since lifetime in PVA is about 60% of that in MMT. At higher PVA loading, an increase in lifetime is observed, interpreted in terms of the presence of micro defects in the exfoliated MMT, since the interlayer space of the exfoliated structure is higher than that in the intercalated structure. The authors find also a small increase in the open spaces in the PVA/MMT structure when including Pd (Figure 7), attributed to the replacement of the intermolecular hydrogen bonding from PVA chains by the interactions between PVA chains and Pd. Figure 7B shows the great sensitivity of the PALS technique in highlighting variations in the sizes of micro-defects well below the tenth of nm.

### 4.4. Bioplastics and Natural Polymers

The importance of natural polymers and bioplastics does not need to be emphasized. Various studies used PALS, combined with other techniques. An investigation concerning the release of a bioactive compound (curcumin) by biopolymers (carrageenan and chitosan) and bioplastics (PLA and poly (butylene adipate-co-terephthalate)) [101] pointed out the role of the free volume. Indeed, a negative correlation has been shown between the release of curcumin and free volume fraction.

Structural changes in a chitosan matrix induced by absorption of two metal ions (Cu (II) and Cr (VI)) were studied using various techniques [102]. o-Ps lifetime is higher in the chitosan matrix than in the samples containing metal ions. The decrease is linear at increasing concentration of Cu, while a strong decrease is observed at low concentrations (<0.38 mM) for the Cr-absorbed samples, which becomes smaller at high concentrations. The results point out that the type of absorbed ion influences the bio matrix more than its concentration, due to the different chitosan-ion interactions: via amino groups in the case of Cu (II), and via both amino and hydroxyl groups in the case of Cr (VI), with a consequent more marked reduction of the free volume holes.

Cellulose esters with aromatic substituents (trifluoromethylbenzoate and methylbenzoate, respectively) were synthesized [103]. The authors found that the free volume is influenced by the different packing and positions of the CH_3_ and CF_3_ groups in the substituents. In particular, the meta positions exhibit the highest number density of free volume holes, which is inversely related to both the universal hardness and indentation elastic modulus of the two esters.

Another study involving cellulose was undertaken by Nuruddin et al. [104]. The authors prepared chiral nematic and shear oriented cellulose nanocrystal films, with the aim to find a possible relationship between free volume and gas barrier performance. Their results show that sheared films have lower free volume and exhibit higher tortuosity than chiral nematic self-assembled films, which hinders the diffusion of gases throughout the films. Cellulose nanocrystal films show a higher barrier performance than high barrier polymer films like poly-vinyl alcohol and ethylene vinyl alcohol.

A large number of other studies involving PALS could be cited about many different polymer structures; we have to limit ourselves to the following, to illustrate other connections between free volume as explored by PALS and structural properties.

Ethylene vinyl acetate (EVA) is used as encapsulant in crystalline Si photovoltaic modules. EVA deacetylation negatively influences the performance of the latter and to clarify the degradation mechanism, a PALS investigation was undertaken [105]. Using an energy-tunable slow positron pulse beam, it was possible to assess that deacetylation (involving a reduction in size of the free volume holes) mainly occurs at the interface between EVA and the other components. It was shown that a reduction of o-Ps lifetime becomes significant after more than 3000 h of UV irradiation, but that a treatment of 3000 h UV irradiation followed by a 1500 h dump heat produces a more marked lifetime reduction than mere UV irradiation. UV irradiation may be the reason for a photochemical reaction, but dump heat conditions may promote the hydrolysis of acetate by infiltrated water. The depth profile of the sizes of free volume holes in an outdoor exposed module was found very similar to that obtained for the sample UV irradiated and subsequently exposed to dump heat, suggesting that the same deacetylation mechanism occurs with outdoor exposure.

**Figure 8:**
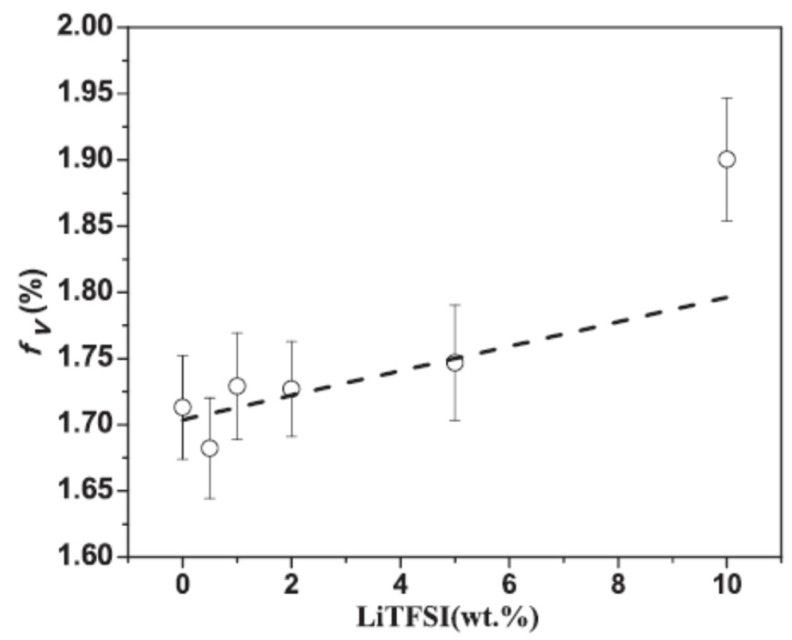
Relative free volume versus the Li salt concentration (reproduced from ref. [106] with permission from Elsevier. DOI: 10.1016/j.ssi.2019.05.025).

Poly (ethylene oxide) (PEO) has been the subject of various investigations [106,107]. The microstructure of the polymer electrolyte PEO-LiTFSI was studied with varying electrolyte concentrations. In particular, an increase of o-Ps lifetime and a small decrease in intensity at increasing concentration was found, which mirrors the number density of holes. The relative free volume increases linearly with the LiTFSI concentration up to 5 wt%, showing a drastic increment at higher loading, in agreement with an analogous behavior of the ionic conductivity (Figure 8).

**Figure 9:**
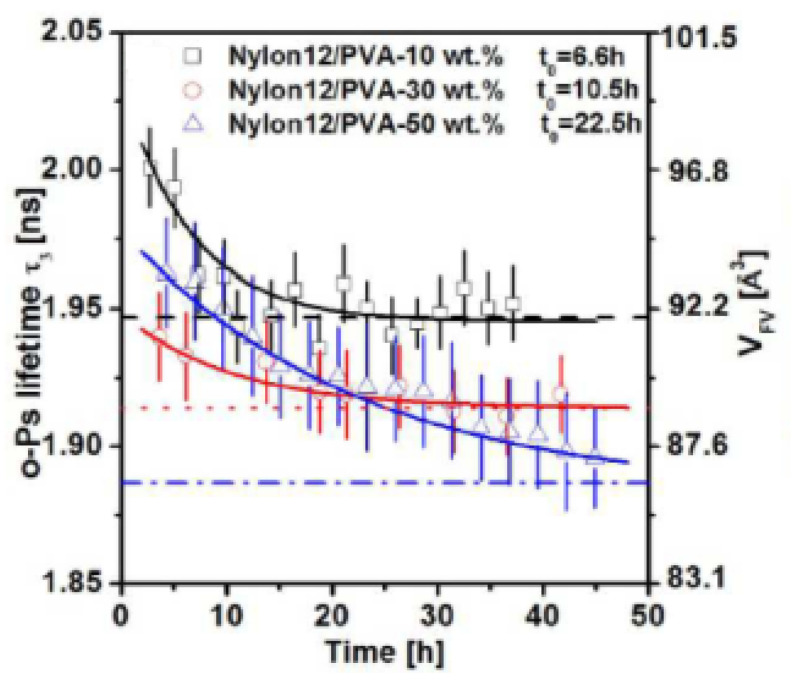
o-Ps lifetime and mean hole free volume for super critical CO_2_ treated Nylon12/PVA blend films at 50 °C and 20 MPa for one hour versus elapsed time after CO_2_ depressurization. The dashed lines, dotted lines, and dashed-dotted lines represent the o-Ps lifetime of the untreated films with the PVA content of 10, 30, and 50 wt%, respectively. The solid lines represent the fitting relaxation curves of the three samples (reproduced from ref. [108] with permission from Acta Physica Polonica A. DOI: 10.12693/APhysPolA.132.1552).

The free volume of blends Nylon12/PVA, treated with supercritical CO_2_, was studied using PALS [108]. Lifetime and intensity of o-Ps decrease with increasing content of PVA with respect to neat Nylon12, which is attributed to the higher crystallinity of PVA as well as the extra intermolecular hydrogen bonding between Nylon12 and PVA, acting as physical crosslinks that reduce the molecular mobility of Nylon12 and the free volume sizes. Treatment with supercritical CO_2_ expands the free volume holes and, after depressurization, o-Ps lifetime reduces exponentially with time, showing relaxation times that are the longer, the higher the concentration of PVA in the blends (Figure 9). This agrees with the previous remark of reduced mobility of Nylon12.

Two methods applied to various classes of polymers are irradiation and photopolymerization. In this case, too, PALS has been used to get insight into the microstructural changes involved in the processes. Here we consider a few examples.

### 4.5. Irradiated Polymers

Polymer irradiation is another important subject, with broad applications. Among recent PALS investigations, we can mention a study on irradiated PE with doses up to 1 MGy [109]. While o-Ps lifetime does not change significantly, o-Ps intensity decreases with the dose. Furthermore, intensity further decreases with time after the maximum doses, which is explained in terms of radicals that form carbonyl groups, enhancing with time and inhibiting Ps formation.

Another study was carried out in PVA/Li_2_B_4_O_7_ polymer films, irradiated with an electron beam [110]. The results show an increase in the free volume hole size with the dose, which is interpreted as a coalescence of smaller holes into bigger ones. This process makes easier the movements of the segmental polymer chains, which undergo chain scissions, and facilitates the motion of Li^+^ ions within the polymer segment. Indeed, an increase in the ionic conductivity with the electron dose is observed.

An investigation on the effect of gamma irradiation on CR-39 has been carried out by Kumar et al. [111]. The goal was to study the microstructural changes involved by irradiation and in particular the free volume. The two main parameters of o-Ps, lifetime and intensity, show an opposite behavior under irradiation: the lifetime (and therefore the size of the hole) decreases, while the intensity increases. On the whole, the free volume increases with irradiation, due to the amorphization of the polymer, in agreement with XRD results.

### 4.6. Photopolymerization

**Figure 10:**
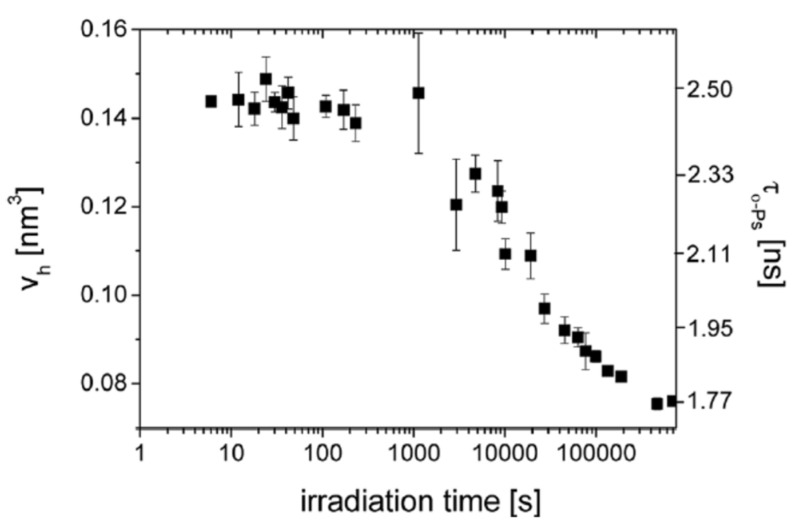
o-Ps lifetime and hole volume versus irradiation time during photopolymerization of SPOT LV (reproduced from ref. [112] with permission from the Royal Society of Chemistry. DOI: 10.1039/c8ra07578f).

The photopolymerization process has also been studied using PALS. Svajdlenkova et al. [112] investigated the evolution of the free volume during photopolymerization in a commercial photopolymer (SPOT LV). Since network formation proceeds at a fast rate, while PALS measurements are ordinarily of the order of at least one hour (depending on the activity of the source), it was necessary to discontinuously irradiate the sample to allow to carry out the PALS spectra during the non-irradiation period. Furthermore, a light source of weak intensity was used to slow down the photopolymerization process. The mean value of the o-Ps lifetime distribution, and therefore the average hole volume, decrease after 1000 s irradiation, pointing out a shrinkage during crosslinking, due to the shortening of the chemical bonds (Figure 10). Intensity of o-Ps versus irradiation shows three regions characterized by different kinetics. After a slow increase, at about 5000 s a change in the slope is observed, corresponding to the acceleration of the curing process. A lower slope above 20,000 s indicates a slowdown of the crosslinking reaction, which is ending. The width of the lifetime distribution also gives interesting information. Indeed, a gradual narrowing after about 5000 s is indicative of the homogenization of the material as the crosslinking proceeds.

Another photopolymerization process inducing volumetric shrinkage has been investigated by means of PALS [113]. Using two commercial dimethacrylate-based dental restorative composites, characterized by a loose and dense packing, the authors found two o-Ps lifetimes, attributed to annihilation in free volume holes of the amorphous phase and annihilation in voids of the crystalline phase for the longest and shorter components, respectively.

### 4.7. Coupling PALS and Dilatometry: An Alternative Route to the Free Volume Fraction

**Figure 11:**
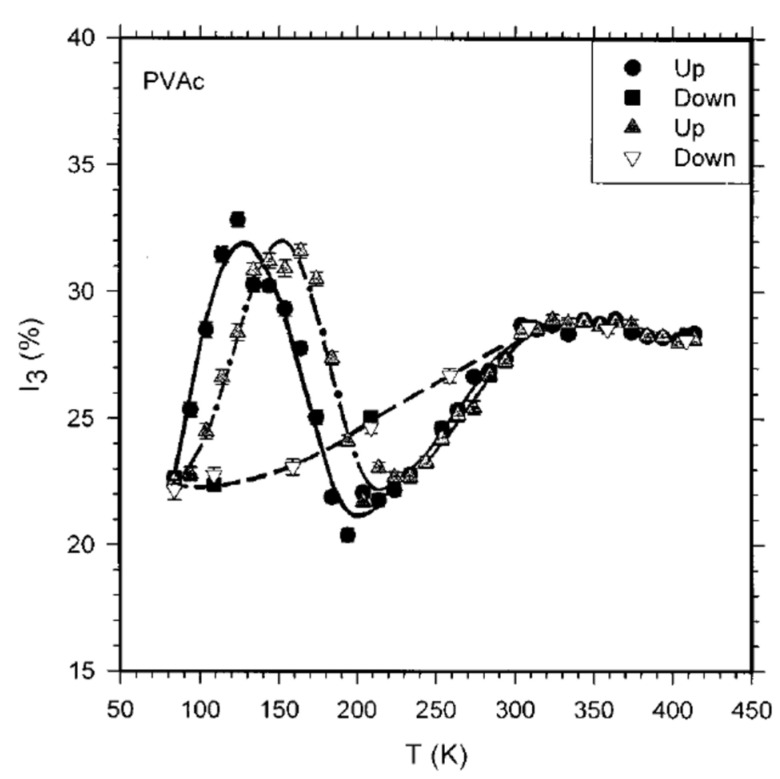
o-Ps intensity versus temperature for PVAc: the full curve is for the first sample (dot and square) and dashed-dotted curve is for the second sample (triangle) during the heating (‘‘Up’’). The dashed curve is drawn through the points obtained during cooling (‘‘Down’’) (reproduced from ref. [115] with permission from American Institute of Physics. DOI: 10.1063/1.475876).

Use of Equation (4) to quantify the free volume fraction is common in many studies but should be cautiously considered. In fact, Ps formation and, consequently, *I*_3_ is influenced by various factors [114,115,116], and it is not easy to disentangle the contribution of the number density of holes. In particular, o-Ps intensity in PVAc samples [115] subjected to wide range of temperatures shows hysteresis (Figure 11).

The effect has been explained in terms of radiation chemistry processes in the terminal track of the positron [117], and in this case, *I*_3_ cannot be simply correlated to the variation in the number density of holes with temperature. Furthermore, it has been observed that *I*_3_ increases in *γ*-irradiated PE and PMMA6N (PMMA containing 6% of methyl acrylate) at low temperature [118]; the effect has been attributed to increased Ps formation on trapped electrons generated by irradiation, therefore not simply related to the number density of holes. A quantitative analysis of this result [119] led to the conclusion that the probability of Ps formation is the main factor influencing *I*_3_ and not simply the number density of free volume holes, since all o-Ps become trapped before annihilation. According to the spur model [117], during their slowing down, positrons ionize the molecules of the medium producing electrons; interaction of the positron with one of the electrons created in its terminal track (spur) can produce Ps. It follows that no Ps can be formed in the lack of the spur electrons, even if free volume holes are present. We should also consider that the presence of positron acceptors (such as carbonyl groups) is also influencing Ps formation and hence the value of *I*_3_. Therefore, Ps intensity is often the result of various complicated and interrelated processes [120].

According to the previous caveats on the use of Ps intensity as a proxy for the number density of free volume holes, *N*, we use a different approach, introduced by Srithawatpong et al. [121] and followed also by other authors [122]. The free volume fraction *f* is:

(6) $$f=\frac{Nv_{h}}{V_{sp}}$$

*V_sp_* is the specific volume, that is, the sum of free volume and the (specific) occupied volume:

(7) $$V_{sp}=Nv_{h}+V_{occ}$$

where *V_occ_* is defined in terms of the Van der Waals volume and the interstitial free volume [122]: *V_occ_ = V_VdW_ + V_if_*. This latter consists of local voids too minute to host even a small probe such as Ps and it is associated with the occupied volume. The dependence of the occupied volume on the temperature is ascribed to the expansion of such interstitial free volumes and incorporated in the lattice-hole model [123]. In Equations (6) and (7), *v_h_* is the average volume of the holes.

**Figure 12:**
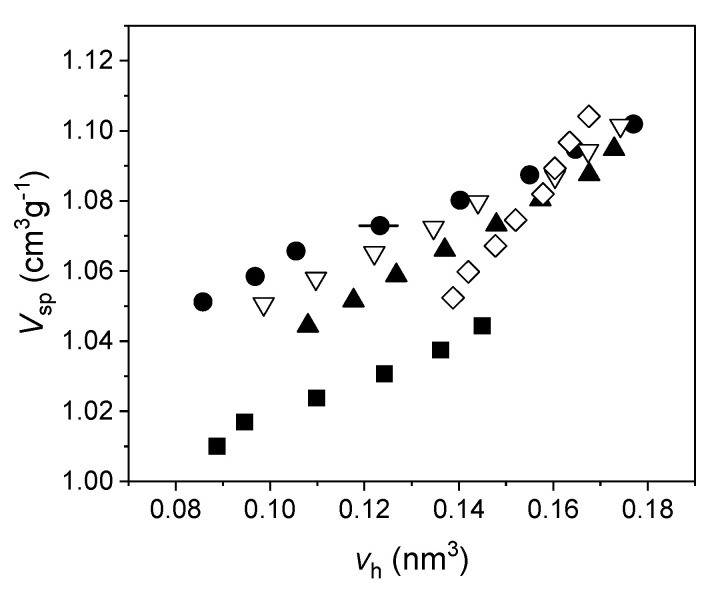
*V*_sp_ versus *v*_h_, using spherical approximation for the holes, in five different polybutadiene-polyisoprene rubbers. Uncertainties for *V*_sp_ are within the size of the data, for *v*_h_ are shown for a single data point for the sake of clarity (reproduced from ref. [124]. DOI: 10.1002/pi.6431).

It is possible to obtain *N* by combining PALS with dilatometry: this technique supplies the specific volume versus temperature (our investigations were carried out at atmospheric pressure), while from PALS, we get the average volume of the hole as a function of the temperature, too. Therefore, by plotting *V_sp_* versus *v_h_* (both evaluated at the same temperature), *N* is the slope (at any temperature) of the curve interpolating the experimental data. If both *V_sp_* and *v_h_* are linear with temperature, *N* = const. An example is given in Figure 12.

By assuming different guesses for the shape of the holes, we get different values of *v_h_* at a given temperature, and therefore *N* is dependent on the choice of the geometry for the cavity. This also influences the values of *f*, which can be compared to the free volume fraction *h*, as supplied by the theory, for instance the Simha-Somcynsky equation of state of polymers. In this connection, we point out the unique role played by dilatometry, since it also allows to find the scaling thermodynamic parameters (temperature and specific volume, in the case the measurements are carried out at atmospheric pressure), specific of each material, to be inserted in the equation of state, in order to evaluate *h* for the investigated polymer. Since the Simha-Somcynsky theory is valid for amorphous polymers at equilibrium, we restricted our investigations to temperatures above the glass transition, although it is possible to also extend the theory in the glassy state [125]. We explored polystyrene with different molecular weight [126], perfluoropolyethers and polypropylene glycols with low molecular weights [127,128], atactic polypropylene [129], polyvinyl acetate [130], a thermoplastic polyurethane [131], and different rubber blends [124,132,133]. Our goal was to explore a possible influence of the investigated structure on the free volume. We adopted a cylindrical geometry for the holes [134], in order to make a comparison with spheres, although we should always have in mind that real holes are irregularly shaped. For a given aspect ratio of the cylinder a different value of the hole volume is obtained, for the same o-Ps lifetime. As a consequence, *f* changes, too, according to Equation (6). Using a least square procedure between *f* and *h* we found, for each investigated polymer, the cylindrical cavity which produced the best fit. We found that generally the spherical shape is less suitable to describe the free volume holes than cylindrical cavities. In particular, for polystyrenes [126] and polyvinyl acetate [130], we found flattened holes, with aspect ratios ranging from 0.25 to 0.54. We remark that the results do not change if we consider, as alternative geometry, parallelepipeds.

Similar findings were found in various elastomers [124,132,133]: in these cases, too, holes are well represented by flattened cylinders. Therefore, it seems that flattened holes are more common than spherical holes, in our investigated polymers.

However, the same procedure mentioned above, as applied to polybutadiene-polyisoprene blends [124] and acrylonitrile-butadiene rubbers [133], as well as a terpolymer acrylonitrile-polybutadiene-polyisoprene [133], did not produce satisfactory fits, for any geometrical choice of the cavity. Cylindrical or prismatic cavities reduce the systematic differences between the free volume fraction supplied by the theory and the same quantity evaluated from PALS and dilatometry, with respect to the spherical approximation; nevertheless, the fits, in terms of statistical test, are not acceptable.

In these cases, we found that the expansion of the holes with temperature seems to proceed anisotropically, being easier in some directions than in others. In other terms, the agreement between *f* and *h* is very good under the guess that the cylinder representing the cavity has a non-linear dependence of the height *s* with the radius *r*:

(8) $$\frac{s}{s_{0}}={\left(\frac{r}{r_{0}}\right)}^{p}$$

**Figure 13:**
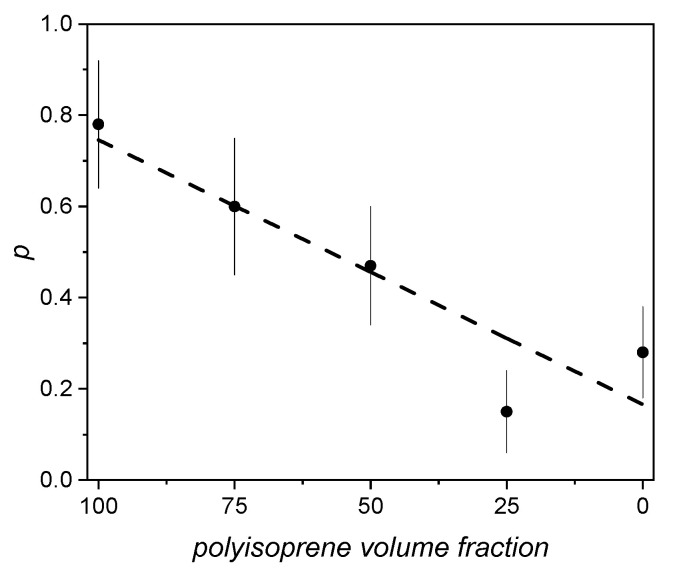
Behavior of parameter *p* versus polyisoprene volume fraction in polybutadiene–polyisoprene blends (reproduced from ref. [133]. DOI: 10.1002/pi.6431).

*s*_0_ and *r*_0_ being height and radius at a given temperature. *p* can be assumed as an index of anisotropic growth: for a cylinder expanding isotropically with temperature *p* = 1. In particular, in polybutadiene-polyisoprene blends we found that *p* decreases almost linearly with the content of polyisoprene volume fraction (Figure 13), while the presence of acrylonitrile in acrylonitrile-butadiene blends does not change, within the errors, the value of *p* found for pure butadiene.

It seems that *p* can have some relation with the structure of the material. An analogous anisotropic expansion of the holes was also found for two classes of oligomers: perfluoropolyethers [127] and polypropylene glycols [128].

We point out that the anisotropic expansion of the holes, although obtained with a simplified shape, is not in contrast with the presence of physical or chemical constraints (e.g., entanglements or crosslinks), which may hinder the macromolecular motions in some particular directions.

Another result has been found in the three investigated butadiene-acrylonitrile rubbers and the terpolymer polyisoprene-polybutadiene-acrylonitrile [133], extrapolating the hole sizes at the glass transition: they are found in the range 0.6–0.7 nm. These values are comparable to the effective bond length *l*, defined as the square root of the ratio between the unperturbed mean-square end-to-end distance ⟨*R*^2^⟩_0_ of a chain and the number of its backbone bonds, which results in 0.5± 0.2 nm, according to Wang [135]. A similar value (0.6 nm) is given by Miller [136]. We found analogous results for a fluoroelastomer and a cis-polyisoprene rubber [132]. In atactic polypropylene [129], the result is 0.56 nm. Also the investigated oligomers show similar values: for polypropylene glycols [128] the size is 0.47–0.48 nm, and for perfluoropolyethers [127] the result is 0.6–0.63 nm. It is impressive this remarkable similarity between a typical size of holes at the glass transition for different polymers and the effective bond length, a parameter related to repetition motions and independent of the macromolecular structure.

## 5. Conclusions

PALS is a valuable technique to investigate the intra- and inter-chain spaces in polymers, with the unique capability to probe holes with sub-nanometric sizes. In the above discussed examples, we tried to point out the importance of this experimental technique that gives a microscopic interpretation of the free volume, a concept related to different macroscopic properties of the material. Indeed, as reported in the introduction, mechanical, thermal, and transport properties of polymeric structures are identified and correlated with the free volume fraction.

Our studies have shown that a combination of PALS and dilatometry results, integrated with the prediction of the Simha-Somcynsky theory, can give insight on the shape of the free volume holes. Although real holes are irregularly shaped, we have seen that in some cases a flattened geometry allows us to get better agreement with the theoretical free volume fraction. Furthermore, the growth of the free volume holes with temperature in some polymers seems to be not isotropic, but the expansion appears to be easier in some directions. This sounds reasonable, when we consider that the segmental motions of the polymer chains may be hindered by constraints of various kinds.

Future investigations will take advantage of the always better performance of digital spectrometers, both in terms of resolution and increased statistics. Advances in speed and memory capability of next generation computers should make accessible computer simulations of free volumes, nowadays limited to a few examples, e.g., [137,138,139,140] due to the large amount of time required. This will open new possibilities to compare the results of the experiments, in addition to the theoretical models.

In conclusion, any advance in the PALS technique, both in the apparatus and data analysis, will allow a more complete view of the free volume fraction, a valuable quantity to explain many polymer properties, but which cannot be measured with macroscopic techniques in a direct way.

## Figures and Tables

**Table 1:** Ps lifetime and intensity of some polymers. The radius of the spherical hole is also shown. LDPE: low density polyethylene, HDPE: high density polyethylene, PMMA: poly (methyl methacrylate), PET: poly (ethylene terephthalate), SBR: styrene/butadiene rubber, PTFE: poly tetrafluoroethylene, PDMS: polydimethylsiloxane, PTMSP: poly [1-(trimethyl-silyl)propine].

Polymer	τ_3_ (ns)	R_3_ (nm)	I_3_ (%)	τ_4_ (ns)	R_4_ (nm)	I_4_ (%)	Ref.
LDPE	2.55 ± 0.01	0.33	21.1 ± 0.4				[38]
HDPE	2.38 ± 0.04	0.32	19.7 ± 0.3				[39]
Nylon-6	1.55 ± 0.02	0.24	24.5 ± 0.04				[40]
PMMA	1.92 ± 0.01	0.28	23.6 ± 0.02				[41]
PET	1.66 ± 0.03	0.25	20.4 ± 0.3				[42]
Cis1,4-polybutadiene	2.614 ± 0.005	0.34	39.45 ± 0.07				[43]
SBR	2.50 ± 0.02	0.33	34.2 ± 0.9				[44]
PDMS	3.27 ± 0.03	0.39	30.3 ± 0.5				[45]
Nafion	3.27 ± 0.01	0.39	6.33 ± 0.06				[46]
PTFE	1.12 ± 0.09	0.19	9.6 ± 0.9	3.92 ± 0.02	0.43	13.8 ± 0.2	[47]
PTMSP	4.7 ± 0.3	0.47	9.7 ± 0.5	13.8 ± 0.1	0.79	31.1 ± 0.6	[48]

## Data Availability

The data presented in Section 4.7 of this study are available on request from the corresponding author.
